# Dimethyl (1-hydr­oxy-1,2-diphenyl­ethyl)phospho­nate

**DOI:** 10.1107/S1600536809029808

**Published:** 2009-07-31

**Authors:** M. Nawaz Tahir, Nurcan Acar, Hamza Yilmaz, Riaz H. Tariq

**Affiliations:** a Department of Physics, University of Sargodha, Sargodha, Pakistan; b Department of Chemistry, Faculty of Science, University of Ankara, Ankara, Turkey; c Institute of Chemical and Pharmaceutical Sciences, The University of Faisalabad, Faisalabad, Pakistan

## Abstract

In the crystal of the title compound, C_16_H_19_O_4_P, the mol­ecules are dimerized with *R*_2_^2^(10) ring motifs through the hydr­oxy and P=O O atoms. The dihedral angle between the aromatic rings is 66.89 (9)°. There are π–π inter­actions [centroid–centroid distance = 3.9669 (16) Å] between the benzene rings of adjacent benzyl groups. A C—H⋯π inter­action between the aromatic rings where C—H is from a benzyl group is also present.

## Related literature

For the preparation and crystal structures of α-hydr­oxy phospho­nates, see: Acar *et al.* (2009*a* ▶,*b* ▶); Tahir *et al.* (2007 ▶, 2009*a* ▶,*b* ▶). For an isomer of the title compouns, see: Acar *et al.* (2009*a* ▶). For ring-motifs, see: Bernstein *et al.* (1995 ▶).
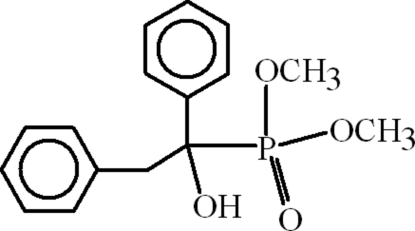


## Experimental

### 

#### Crystal data

C_16_H_19_O_4_P
*M* *_r_* = 306.28
Monoclinic, 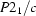

*a* = 8.4767 (5) Å
*b* = 15.8978 (10) Å
*c* = 13.3888 (7) Å
β = 119.397 (3)°
*V* = 1571.97 (17) Å^3^
*Z* = 4
Mo *K*α radiation
μ = 0.19 mm^−1^
*T* = 296 K
0.25 × 0.18 × 0.15 mm

#### Data collection

Bruker Kappa APEXII CCD diffractometer
Absorption correction: multi-scan (*SADABS*; Bruker, 2005 ▶) *T*_min_ = 0.963, *T*_max_ = 0.974
16757 measured reflections
3861 independent reflections
2183 reflections with *I* > 2σ(*I*)
*R*_int_ = 0.043

#### Refinement

*R*[*F* ^2^ > 2σ(*F* ^2^)] = 0.047
*wR*(*F* ^2^) = 0.126
*S* = 1.01
3861 reflections
195 parameters
H atoms treated by a mixture of independent and constrained refinement
Δρ_max_ = 0.27 e Å^−3^
Δρ_min_ = −0.28 e Å^−3^

### 

Data collection: *APEX2* (Bruker, 2007 ▶); cell refinement: *SAINT* (Bruker, 2007 ▶); data reduction: *SAINT*; program(s) used to solve structure: *SHELXS97* (Sheldrick, 2008 ▶); program(s) used to refine structure: *SHELXL97* (Sheldrick, 2008 ▶); molecular graphics: *ORTEP-3 for Windows* (Farrugia, 1997 ▶) and *PLATON* (Spek, 2009 ▶); software used to prepare material for publication: *WinGX* (Farrugia, 1999 ▶) and *PLATON* .

## Supplementary Material

Crystal structure: contains datablocks global, I. DOI: 10.1107/S1600536809029808/at2852sup1.cif

Structure factors: contains datablocks I. DOI: 10.1107/S1600536809029808/at2852Isup2.hkl

Additional supplementary materials:  crystallographic information; 3D view; checkCIF report

## Figures and Tables

**Table 1:** Hydrogen-bond geometry (Å, °)

*D*—H⋯*A*	*D*—H	H⋯*A*	*D*⋯*A*	*D*—H⋯*A*
O1—H1⋯O2^i^	0.79 (2)	1.92 (2)	2.684 (2)	164 (3)
C12—H12⋯*CgA*^ii^	0.93	2.86	3.755 (4)	163

Symmetry codes: (i) 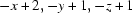; (ii) 
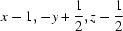
. *CgA* is the centroid of the C1–C6 ring.

**Fig. 1.**
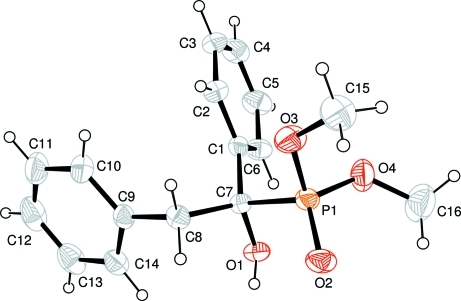
View of the title compound with the atom numbering scheme. The thermal ellipsoids are drawn at the 50% probability level. H-atoms are shown by small circles of arbitrary radii.

**Fig. 2.**
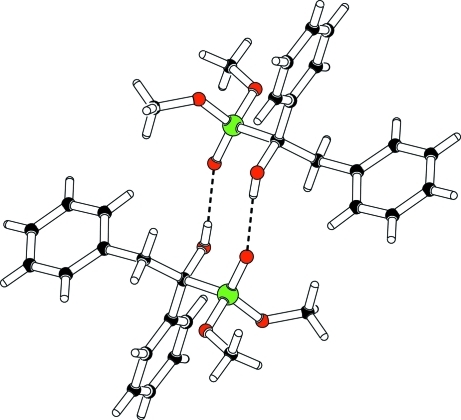
The partial packing (PLATON; Spek, 2009) which shows that molecules form dimers.

## supplementary crystallographic information

### Comment

We have reported preparation and crystal structures containing α-Hydroxy phosphonates (Tahir *et al.*, 2007, 2009*a*,*b*) and (Acar *et al.* 2009*a*,*b*). The title compound (I, Fig. 1) is chemically isomer of (II) (Acar *et al.*, 2009*a)*. The crystals of title compound were selected from the sample of Acar *et al.*, 2009*a* present at low yield.

The difference of (I) with (II) exist due to various reasons. In (I) the dihedral angle between the aromatic rings A (C1–C6) and B (C9–C14) is 66.89 (9)° compared to 72.28 (11)°. The distorted tetrahedral geometry around C7 have range of angles 104.15 (13)° to 113.43 (16)° instead of 104.4 (2)° to 112.8 (2)°. There exist small variations in bond lengths e.g, P1—C7 is 1.835 (2) Å compared to 1.845 (3) Å. In (I) the molecules form dimers only and dimers are not linked to each other as observed in (II). The dimers (Fig. 2) are formed due to intermolecular H-bonding of O—H···O type with ring motif *R*_2_^2^(10) (Bernstein *et al.*, 1995). There exist C—H···π interaction between the two aromatic rings (Table 1) and π–π interaction at a distance of 3.9669 (16) Å between the centroids of aromatic ring B with CgB···CgB^i^ [symmetry code: (i) 1 - *x*, 1 - *y*, -*z*].

### Experimental

The preparation is reported in Acar *et al.*, 2009*a*.

### Refinement

The H-atom of hydroxy group were refined freely. The other H-atoms were positioned geometrically, with C—H = 0.93, 0.96 and 0.97 Å for aromatic, methyl and methylene H, and constrained to ride on their parent atoms, with U_iso_(H) = xU_eq_(C,O), where x = 1.5 for methyl H, and x = 1.2 for all other H atoms.

### Crystal data

**Table:**

C_16_H_19_O_4_P	*F*(000) = 648
*M**_r_* = 306.28	*D*_x_ = 1.294 Mg m^−^^3^
Monoclinic, *P*2_1_/*c*	Mo *K*α radiation, λ = 0.71073 Å
Hall symbol: -P 2ybc	Cell parameters from 3861 reflections
*a* = 8.4767 (5) Å	θ = 2.8–28.3°
*b* = 15.8978 (10) Å	µ = 0.19 mm^−^^1^
*c* = 13.3888 (7) Å	*T* = 296 K
β = 119.397 (3)°	Prismatic, colourless
*V* = 1571.97 (17) Å^3^	0.25 × 0.18 × 0.15 mm
*Z* = 4	

### Data collection

**Table:**

Bruker Kappa APEXII CCD diffractometer	3861 independent reflections
Radiation source: fine-focus sealed tube	2183 reflections with *I* > 2σ(*I*)
graphite	*R*_int_ = 0.043
Detector resolution: 7.40 pixels mm^-1^	θ_max_ = 28.3°, θ_min_ = 2.8°
ω scans	*h* = −11→10
Absorption correction: multi-scan (*SADABS*; Bruker, 2005)	*k* = −21→21
*T*_min_ = 0.963, *T*_max_ = 0.974	*l* = −17→17
16757 measured reflections	

### Refinement

**Table:**

Refinement on *F*^2^	Primary atom site location: structure-invariant direct methods
Least-squares matrix: full	Secondary atom site location: difference Fourier map
*R*[*F*^2^ > 2σ(*F*^2^)] = 0.047	Hydrogen site location: inferred from neighbouring sites
*wR*(*F*^2^) = 0.126	H atoms treated by a mixture of independent and constrained refinement
*S* = 1.01	*w* = 1/[σ^2^(*F*_o_^2^) + (0.0517*P*)^2^ + 0.2804*P*] where *P* = (*F*_o_^2^ + 2*F*_c_^2^)/3
3861 reflections	(Δ/σ)_max_ < 0.001
195 parameters	Δρ_max_ = 0.27 e Å^−^^3^
0 restraints	Δρ_min_ = −0.28 e Å^−^^3^

### Special details

**Table:**

Geometry. Bond distances, angles *etc*. have been calculated using the rounded fractional coordinates. All su's are estimated from the variances of the (full) variance-covariance matrix. The cell e.s.d.'s are taken into account in the estimation of distances, angles and torsion angles
Refinement. Refinement of *F*^2^ against ALL reflections. The weighted *R*-factor *wR* and goodness of fit *S* are based on *F*^2^, conventional *R*-factors *R* are based on *F*, with *F* set to zero for negative *F*^2^. The threshold expression of *F*^2^ > σ(*F*^2^) is used only for calculating *R*-factors(gt) *etc*. and is not relevant to the choice of reflections for refinement. *R*-factors based on *F*^2^ are statistically about twice as large as those based on *F*, and *R*- factors based on ALL data will be even larger.

### Fractional atomic coordinates and isotropic or equivalent isotropic displacement parameters (Å^2^)

**Table:**

	*x*	*y*	*z*	*U*_iso_*/*U*_eq_	
P1	1.16204 (7)	0.38185 (3)	0.45831 (5)	0.0440 (2)	
O1	0.83863 (19)	0.37902 (9)	0.43056 (13)	0.0455 (5)	
O2	1.20677 (18)	0.46360 (9)	0.51545 (14)	0.0587 (5)	
O3	1.25054 (19)	0.36505 (11)	0.38159 (14)	0.0660 (6)	
O4	1.2208 (2)	0.30558 (10)	0.54132 (14)	0.0762 (6)	
C1	0.8814 (2)	0.27486 (12)	0.31946 (16)	0.0393 (6)	
C2	0.9200 (3)	0.24523 (14)	0.23689 (18)	0.0528 (8)	
C3	0.8833 (3)	0.16197 (17)	0.2000 (2)	0.0636 (9)	
C4	0.8104 (3)	0.10853 (16)	0.2456 (2)	0.0684 (9)	
C5	0.7733 (3)	0.13707 (15)	0.3286 (2)	0.0658 (9)	
C6	0.8080 (3)	0.21952 (13)	0.3647 (2)	0.0512 (8)	
C7	0.9192 (2)	0.36591 (12)	0.36066 (16)	0.0371 (6)	
C8	0.8505 (3)	0.43043 (13)	0.26211 (17)	0.0444 (7)	
C9	0.6550 (3)	0.41928 (13)	0.17226 (18)	0.0456 (7)	
C10	0.6074 (4)	0.38734 (16)	0.0652 (2)	0.0691 (9)	
C11	0.4283 (5)	0.37485 (19)	−0.0165 (2)	0.0922 (11)	
C12	0.2939 (4)	0.39384 (18)	0.0079 (3)	0.0883 (11)	
C13	0.3378 (3)	0.42615 (17)	0.1120 (3)	0.0762 (10)	
C14	0.5166 (3)	0.43991 (14)	0.1941 (2)	0.0565 (8)	
C15	1.4435 (3)	0.36510 (19)	0.4291 (3)	0.0795 (11)	
C16	1.3067 (5)	0.3068 (2)	0.6594 (3)	0.1003 (15)	
H1	0.844 (3)	0.4272 (14)	0.4467 (19)	0.0546*	
H2	0.97088	0.28115	0.20561	0.0634*	
H3	0.90891	0.14285	0.14384	0.0763*	
H4	0.78583	0.05302	0.22077	0.0821*	
H5	0.72445	0.10061	0.36055	0.0789*	
H6	0.78141	0.23814	0.42066	0.0615*	
H8A	0.86629	0.48651	0.29419	0.0533*	
H8B	0.92463	0.42629	0.22556	0.0533*	
H10	0.69787	0.37396	0.04765	0.0829*	
H11	0.39936	0.35349	−0.08820	0.1106*	
H12	0.17327	0.38469	−0.04633	0.1061*	
H13	0.24629	0.43934	0.12862	0.0914*	
H14	0.54368	0.46321	0.26443	0.0678*	
H15A	1.48869	0.42133	0.45095	0.1196*	
H15B	1.47329	0.34472	0.37294	0.1196*	
H15C	1.49759	0.32926	0.49535	0.1196*	
H16A	1.42483	0.28223	0.68969	0.1505*	
H16B	1.23695	0.27518	0.68516	0.1505*	
H16C	1.31802	0.36388	0.68549	0.1505*	

### Atomic displacement parameters (Å^2^)

**Table:**

	*U*^11^	*U*^22^	*U*^33^	*U*^12^	*U*^13^	*U*^23^
P1	0.0404 (3)	0.0400 (3)	0.0461 (3)	0.0007 (2)	0.0169 (2)	−0.0084 (3)
O1	0.0601 (9)	0.0362 (8)	0.0526 (9)	−0.0011 (7)	0.0373 (8)	−0.0051 (7)
O2	0.0475 (8)	0.0454 (9)	0.0735 (10)	−0.0043 (7)	0.0221 (8)	−0.0213 (8)
O3	0.0412 (8)	0.0905 (13)	0.0680 (10)	−0.0029 (8)	0.0282 (8)	−0.0230 (9)
O4	0.0837 (12)	0.0515 (11)	0.0533 (10)	0.0049 (8)	0.0027 (9)	0.0020 (8)
C1	0.0361 (10)	0.0373 (11)	0.0383 (11)	0.0022 (8)	0.0134 (9)	−0.0029 (9)
C2	0.0604 (13)	0.0521 (14)	0.0467 (12)	0.0047 (10)	0.0268 (11)	−0.0044 (11)
C3	0.0687 (16)	0.0608 (17)	0.0498 (14)	0.0114 (12)	0.0203 (12)	−0.0182 (13)
C4	0.0593 (14)	0.0453 (15)	0.0803 (19)	−0.0029 (11)	0.0185 (14)	−0.0198 (14)
C5	0.0632 (15)	0.0430 (14)	0.0912 (19)	−0.0100 (11)	0.0380 (15)	−0.0106 (14)
C6	0.0529 (12)	0.0425 (13)	0.0643 (14)	−0.0049 (10)	0.0334 (11)	−0.0079 (11)
C7	0.0403 (10)	0.0355 (11)	0.0379 (10)	−0.0005 (8)	0.0211 (9)	−0.0020 (9)
C8	0.0472 (12)	0.0397 (12)	0.0494 (12)	−0.0004 (9)	0.0261 (10)	0.0038 (10)
C9	0.0516 (12)	0.0355 (12)	0.0430 (12)	0.0005 (9)	0.0181 (10)	0.0072 (10)
C10	0.0815 (18)	0.0677 (17)	0.0464 (14)	0.0126 (14)	0.0223 (13)	0.0061 (13)
C11	0.108 (2)	0.074 (2)	0.0480 (16)	0.0089 (18)	0.0023 (17)	−0.0025 (15)
C12	0.0672 (18)	0.0561 (18)	0.084 (2)	−0.0079 (14)	−0.0073 (16)	0.0017 (16)
C13	0.0484 (14)	0.0614 (18)	0.096 (2)	0.0007 (12)	0.0179 (14)	0.0064 (16)
C14	0.0518 (13)	0.0459 (14)	0.0631 (15)	0.0036 (10)	0.0215 (12)	0.0023 (12)
C15	0.0484 (14)	0.101 (2)	0.093 (2)	−0.0057 (14)	0.0377 (14)	−0.0070 (17)
C16	0.121 (3)	0.102 (3)	0.0607 (18)	0.000 (2)	0.0312 (19)	0.0099 (18)

### Geometric parameters (Å, °)

**Table:**

P1—O2	1.4606 (16)	C12—C13	1.355 (5)
P1—O3	1.5639 (19)	C13—C14	1.385 (4)
P1—O4	1.5524 (17)	C2—H2	0.9300
P1—C7	1.835 (2)	C3—H3	0.9300
O1—C7	1.419 (3)	C4—H4	0.9300
O3—C15	1.435 (4)	C5—H5	0.9300
O4—C16	1.378 (4)	C6—H6	0.9300
O1—H1	0.79 (2)	C8—H8A	0.9700
C1—C6	1.378 (3)	C8—H8B	0.9700
C1—C7	1.526 (3)	C10—H10	0.9300
C1—C2	1.381 (3)	C11—H11	0.9300
C2—C3	1.394 (3)	C12—H12	0.9300
C3—C4	1.359 (4)	C13—H13	0.9300
C4—C5	1.372 (4)	C14—H14	0.9300
C5—C6	1.378 (3)	C15—H15A	0.9600
C7—C8	1.542 (3)	C15—H15B	0.9600
C8—C9	1.506 (3)	C15—H15C	0.9600
C9—C14	1.381 (4)	C16—H16A	0.9600
C9—C10	1.381 (3)	C16—H16B	0.9600
C10—C11	1.382 (5)	C16—H16C	0.9600
C11—C12	1.364 (6)		
			
O1···O2	3.059 (2)	C5···H16A^v^	2.9000
O1···O4	3.056 (3)	C6···H16A^v^	2.9300
O1···C14	3.151 (3)	C8···H2	2.8300
O1···C15^i^	3.347 (4)	C13···H5^vii^	2.8800
O1···O2^ii^	2.684 (2)	C14···H15B^i^	3.0000
O2···O1^ii^	2.684 (2)	H1···O2	2.80 (3)
O2···O1	3.059 (2)	H1···H8A	2.3400
O3···C2	3.147 (3)	H1···H14	2.5800
O4···C6	3.404 (3)	H1···O2^ii^	1.92 (2)
O4···C12^iii^	3.303 (3)	H2···O3	2.7300
O4···O1	3.056 (3)	H2···C8	2.8300
O1···H6	2.2800	H2···H8B	2.3800
O1···H14	2.7400	H2···H16B^viii^	2.5600
O1···H15B^i^	2.8500	H3···O1^viii^	2.6400
O1···H3^iv^	2.6400	H5···C13^ix^	2.8800
O2···H1	2.80 (3)	H5···H13^ix^	2.5700
O2···H1^ii^	1.92 (2)	H6···O1	2.2800
O2···H16C	2.5500	H8A···H1	2.3400
O2···H14^ii^	2.9000	H8A···H14	2.5900
O3···H8B	2.7000	H8B···O3	2.7000
O3···H2	2.7300	H8B···C2	2.8800
O4···H15C	2.7300	H8B···H2	2.3800
C1···C10	3.524 (3)	H8B···H10	2.3600
C2···O3	3.147 (3)	H10···C2	3.0800
C2···C9	3.398 (3)	H10···H8B	2.3600
C2···C10	3.381 (4)	H12···C4^v^	2.9700
C5···C16^v^	3.574 (5)	H12···C5^v^	2.9700
C6···O4	3.404 (3)	H13···H5^vii^	2.5700
C9···C2	3.398 (3)	H14···O1	2.7400
C10···C2	3.381 (4)	H14···H1	2.5800
C10···C1	3.524 (3)	H14···H8A	2.5900
C12···O4^v^	3.303 (3)	H14···O2^ii^	2.9000
C14···O1	3.151 (3)	H15B···O1^vi^	2.8500
C15···O1^vi^	3.347 (4)	H15B···C14^vi^	3.0000
C16···C5^iii^	3.574 (5)	H15C···O4	2.7300
C2···H10	3.0800	H15C···C3^iii^	3.0700
C2···H8B	2.8800	H16A···C5^iii^	2.9000
C3···H15C^v^	3.0700	H16A···C6^iii^	2.9300
C4···H12^iii^	2.9700	H16B···H2^iv^	2.5600
C5···H12^iii^	2.9700	H16C···O2	2.5500
			
O2—P1—O3	114.27 (10)	C4—C3—H3	120.00
O2—P1—O4	114.23 (9)	C3—C4—H4	120.00
O2—P1—C7	114.08 (10)	C5—C4—H4	120.00
O3—P1—O4	104.36 (10)	C4—C5—H5	120.00
O3—P1—C7	103.91 (9)	C6—C5—H5	120.00
O4—P1—C7	104.81 (10)	C1—C6—H6	119.00
P1—O3—C15	121.25 (18)	C5—C6—H6	119.00
P1—O4—C16	127.83 (17)	C7—C8—H8A	109.00
C7—O1—H1	109.9 (19)	C7—C8—H8B	109.00
C2—C1—C6	118.02 (19)	C9—C8—H8A	109.00
C6—C1—C7	120.45 (18)	C9—C8—H8B	109.00
C2—C1—C7	121.52 (18)	H8A—C8—H8B	108.00
C1—C2—C3	120.6 (2)	C9—C10—H10	119.00
C2—C3—C4	120.4 (2)	C11—C10—H10	119.00
C3—C4—C5	119.5 (2)	C10—C11—H11	120.00
C4—C5—C6	120.3 (2)	C12—C11—H11	120.00
C1—C6—C5	121.2 (2)	C11—C12—H12	120.00
P1—C7—C1	110.58 (13)	C13—C12—H12	120.00
P1—C7—C8	109.76 (15)	C12—C13—H13	119.00
O1—C7—C1	107.11 (15)	C14—C13—H13	119.00
O1—C7—C8	111.40 (17)	C9—C14—H14	120.00
C1—C7—C8	113.43 (16)	C13—C14—H14	120.00
P1—C7—O1	104.15 (13)	O3—C15—H15A	109.00
C7—C8—C9	114.24 (19)	O3—C15—H15B	109.00
C8—C9—C10	121.2 (3)	O3—C15—H15C	109.00
C10—C9—C14	117.5 (2)	H15A—C15—H15B	110.00
C8—C9—C14	121.3 (2)	H15A—C15—H15C	109.00
C9—C10—C11	121.5 (3)	H15B—C15—H15C	109.00
C10—C11—C12	120.0 (3)	O4—C16—H16A	109.00
C11—C12—C13	119.4 (3)	O4—C16—H16B	109.00
C12—C13—C14	121.2 (3)	O4—C16—H16C	109.00
C9—C14—C13	120.5 (2)	H16A—C16—H16B	110.00
C1—C2—H2	120.00	H16A—C16—H16C	109.00
C3—C2—H2	120.00	H16B—C16—H16C	109.00
C2—C3—H3	120.00		
			
O2—P1—O3—C15	59.8 (2)	C2—C1—C7—C8	47.6 (3)
O4—P1—O3—C15	−65.7 (2)	C6—C1—C7—P1	103.4 (2)
C7—P1—O3—C15	−175.28 (19)	C6—C1—C7—O1	−9.5 (3)
O2—P1—O4—C16	0.5 (3)	C6—C1—C7—C8	−132.8 (2)
O3—P1—O4—C16	126.0 (3)	C1—C2—C3—C4	−0.6 (4)
C7—P1—O4—C16	−125.1 (3)	C2—C3—C4—C5	−0.1 (4)
O2—P1—C7—O1	−56.34 (15)	C3—C4—C5—C6	0.6 (4)
O2—P1—C7—C1	−171.09 (13)	C4—C5—C6—C1	−0.5 (4)
O2—P1—C7—C8	63.03 (17)	P1—C7—C8—C9	176.07 (16)
O3—P1—C7—O1	178.59 (12)	O1—C7—C8—C9	−69.1 (2)
O3—P1—C7—C1	63.84 (15)	C1—C7—C8—C9	51.8 (3)
O3—P1—C7—C8	−62.04 (16)	C7—C8—C9—C10	−106.8 (2)
O4—P1—C7—O1	69.34 (14)	C7—C8—C9—C14	72.6 (3)
O4—P1—C7—C1	−45.42 (15)	C8—C9—C10—C11	178.2 (2)
O4—P1—C7—C8	−171.30 (14)	C14—C9—C10—C11	−1.2 (4)
C6—C1—C2—C3	0.8 (3)	C8—C9—C14—C13	−177.5 (2)
C7—C1—C2—C3	−179.6 (2)	C10—C9—C14—C13	2.0 (3)
C2—C1—C6—C5	−0.2 (3)	C9—C10—C11—C12	−0.3 (4)
C7—C1—C6—C5	−179.9 (2)	C10—C11—C12—C13	1.0 (4)
C2—C1—C7—P1	−76.2 (2)	C11—C12—C13—C14	−0.2 (4)
C2—C1—C7—O1	170.90 (19)	C12—C13—C14—C9	−1.3 (4)

Symmetry codes: (i) *x*−1, *y*, *z*; (ii) −*x*+2, −*y*+1, −*z*+1; (iii) *x*+1, −*y*+1/2, *z*+1/2; (iv) *x*, −*y*+1/2, *z*+1/2; (v) *x*−1, −*y*+1/2, *z*−1/2; (vi) *x*+1, *y*, *z*; (vii) −*x*+1, *y*+1/2, −*z*+1/2; (viii) *x*, −*y*+1/2, *z*−1/2; (ix) −*x*+1, *y*−1/2, −*z*+1/2.

### Hydrogen-bond geometry (Å, °)

**Table:**

*D*—H···*A*	*D*—H	H···*A*	*D*···*A*	*D*—H···*A*
O1—H1···O2^ii^	0.79 (2)	1.92 (2)	2.684 (2)	164 (3)
C6—H6···O1	0.93	2.28	2.655 (3)	103.00
C16—H16C···O2	0.96	2.55	3.007 (4)	110.00
C12—H12···CgA^v^	0.93	2.86	3.755 (4)	163.00

Symmetry codes: (ii) −*x*+2, −*y*+1, −*z*+1; (v) *x*−1, −*y*+1/2, *z*−1/2.
